# First data on microflora of loggerhead sea turtle (*Caretta caretta*) nests from the coastlines of Sicily

**DOI:** 10.1242/bio.045252

**Published:** 2020-01-29

**Authors:** Delia Gambino, Maria Flaminia Persichetti, Antonino Gentile, Marco Arculeo, Giulia Visconti, Vittoria Currò, Giulia Caracappa, Daniela Crucitti, Antonio Piazza, Francesca Mancianti, Simona Nardoni, Domenico Vicari, Santo Caracappa

**Affiliations:** 1 Istituto Zooprofilattico Sperimentale della Sicilia “A. Mirri”, Area Territoriale Palermo, Via Gino Marinuzzi 3, Palermo 90129, Italy; 2 Centro di Referenza Nazionale sul benessere, monitoraggio e diagnostica delle malattie delle tartarughe marine, Istituto Zooprofilattico Sperimentale della Sicilia “A. Mirri”, Via Gino Marinuzzi 3, Palermo 90129, Italy; 3 Università di Palermo, Dipartimento STEBICEF, Via Archirafi 28, Palermo 90123, Italy; 4 Area Marina Protetta Isole Pelagie, Via Cameroni, 92031 Lampedusa (AG), Italy; 5 Centro Recupero Fauna Selvatica Bosco di Ficuzza, Via del Bosco 1, Ficuzza di Corleone (PA) 90034, Italy; 6 Department of Veterinary Sciences, University of Pisa, Via Livornese, San Piero a Grado, Pisa 56124, Italy; 7 Department of Veterinary Sciences, University of Messina, Polo Universitario Annunziata, Messina 98100, Italy

**Keywords:** *Caretta caretta*, *Fusarium*, Mediterranean Sea, Microflora, Sea turtle eggs

## Abstract

*Caretta caretta* is threatened by many dangers in the Mediterranean basin, but most are human-related. The purposes of this research were: (i) to investigate microflora in samples from six loggerhead sea turtle nests located on the Sicilian coast and (ii) to understand microbial diversity associated with nests, with particular attention to bacteria and fungi involved in failed hatchings. During the 2016 and 2018 summers, 456 eggs and seven dead hatchling from six nests were collected. We performed bacteriological and mycological analyses on 88 egg samples and seven dead hatchlings, allowing us to isolate: *Fusarium* spp. (80.6%), *Aeromonas hydrophila* (55.6%), *Aspergillus* spp. (27.2%) and *Citrobacter freundii* (9%). Two *Fusarium* species were identified by microscopy and were confirmed by PCR and internal transcribed spacer sequencing. Statistical analyses showed significant differences between nests and the presence/absence of microflora, whereas no significant differences were observed between eggs and nests. This is the first report that catalogues microflora from *C. caretta* nests/eggs in the Mediterranean Sea and provides key information on potential pathogens that may affect hatching success. Moreover, our results suggest the need for wider investigations over extensive areas to identify other microflora, and to better understand hatching failures and mortality related to microbial contamination in this important turtle species.

## Abstract (precis)

**Summary:** We report for the first time the microflora of six loggerhead sea turtle nests on the Sicilian coast, and associated failed hatchings.

## INTRODUCTION

The loggerhead sea turtle (*Caretta caretta*) is a vulnerable species according to the International Union for Conservation of Nature (IUCN) and is included as a protected species under different international conventions [e.g. the Barcelona Convention, the Bern Convention and the Convention on International Trade in Endangered Species of Wild Fauna and Flora (CITES)]. Although *C. caretta* is threatened by many dangers, most are related to human interactions, however other, indirect threats are present; ingestion of fishing hooks or plastic can seriously damage the animal's gastrointestinal tract and impact with boats, accidental capture in fishing nets and the influence of beach tourism on habitats and nesting sites all present threats (Caracappa et al., 2018; Mingozzi et al., 2008). The loggerhead sea turtle is the only sea turtle species that nests along the Eastern Sicilian coastline and on the Lampedusa and Linosa Islands. In previous years, loggerhead nesting sites were recorded on the coasts of Sicily, Sardinia, Apulia and the Ionic coasts of Basilicata and Calabria regions (www.lifegate.it). However, at the national level, nesting is considered sporadic, except for the Ionic sections of the southern Calabria and Pelagian Islands (Linosa and Lampedusa), where loggerhead nesting sites were confirmed. In previous years, there have been significant increases in the numbers of nests recorded along Italian shores; this reached 70 nests in the summer of 2018, along with increases in deposition sites in Sicily. On the Sicilian island, not only has there been increased numbers of nests reported, but also increased involvement of several coastal areas in addition to the Pelagian Islands where nesting has always occurred (www.legambiente.it).

It is important to note that loggerhead survival, egg development and carapacial abnormalities are influenced by environmental factors, such as temperature, humidity, distance from the sea, tidal flow, rain levels and sediment granulometry (Baran et al., 2001; Caracappa et al., 2016; Durmuş et al., 2011; Santoro et al., 2006). These environmental parameters or conditions can cause or encourage bacterial and fungal proliferation, affecting correct embryonic development and thereby causing hatching failures. Indeed, turtle nests, thanks to the presence of nutrients, high temperatures and high humidity, are ideal habitats for microbial growth, and these factors can impact on hatching success by altering nest temperatures and oxygen content (Bézy et al., 2015; Keene et al., 2014).

Several microorganisms have been identified and isolated from unhatched turtle eggs and some are considered important causes of nest mortality (Bailey et al., 2018; Sarmiento-Ramírez et al., 2010). Bacteria belonging to the genus *Vibrio* have been isolated from cloacal swabs of *Lepidochelys olivacea* and *Chelonia mydas agassizii* turtle nests in Costa Rica and Mexico (Acuña et al., 1999; Zavala-Norzagaray et al., 2015). The presence of Enterobacteriaceae, such as *Escherichia coli*, *Salmonella* spp., *Enterobacter* spp., *Klebsiella oxytoca*, *Klebsiella pneumoniae*, *Citrobacter* spp., *Serratia* spp., *Pseudomonas* spp. and *Aeromonas* spp. have been found in *C. mydas* and *L. olivacea* eggs (Al-Bahry et al., 2009; Keene et al., 2014; Santoro et al., 2006). In terms of Gram-positive bacteria, the most frequently isolated species of staphylococci from *C. mydas* nests in Costa Rica were *Staphylococcus aureus*, *Staphylococcus intermedius*, *Staphylococcus epidermidis* and *Staphylococcus cromogenes* (Santoro et al., 2006). Those fungal species isolated from sea turtle nests and eggs come from *Aspergillus*, *Fusarium*, *Chrysosporium*, *Penicillium*, *Emericella*, *Rhizopus*, *Actinomucor* and *Apophysomyces* genera (Bailey et al., 2018; Candan, 2018; Güçlü et al., 2010; Phillott et al., 2002). These genera are predominantly saprophytic species that become opportunistic pathogens under particular conditions, e.g. developing embryo conditions. In particular, *Fusarium solani* and *Fusarium oxysporum* have been reported in the nests of different turtle species in Turkey, Costa Rica, Australia, Brazil, Cape Verde and Italy (Güçlü et al., 2010; Keene et al., 2014; Neves et al., 2015; Phillott and Parmenter, 2014). *Fusarium falciforme* and *Fusarium keratoplasticum* have also been reported in loggerhead sea turtle nests in the USA (Bailey et al., 2018). According to some studies (Phillott, 2004; Sarmiento-Ramírez et al., 2010; and references therein), fungal species belonging to the *Fusarium* genus are the leading cause of hatching failure in turtle eggs. These fungal species target eggs located at the top and sides of nests, at positions in close contact with the surrounding sand. After penetrating the inorganic and organic shell layers, these fungal species reduce respiratory gas exchange, decrease the availability of eggshell calcium for developing embryos and exploit embryonic tissue as nutrient sources (Phillott, 2004).

Currently in the Mediterranean Sea, there is a dearth of information on the microbial contamination of eggs/nests of *C. caretta* and their possible implications for egg hatching. The aims of this study were to investigate the microflora in six loggerhead sea turtle nests located on the Sicilian coast, and to better understand microbial diversity associated with these nests, paying particular attention to bacterial and fungal species as potential causes of hatching failures.

## RESULTS

During our study, 88 samples from six nests were analysed to provide data on the microflora of loggerhead sea turtle nests located on the Sicilian coast. The results of bacteriological and mycological investigations are summarised in Table 1.

**Table 1.**
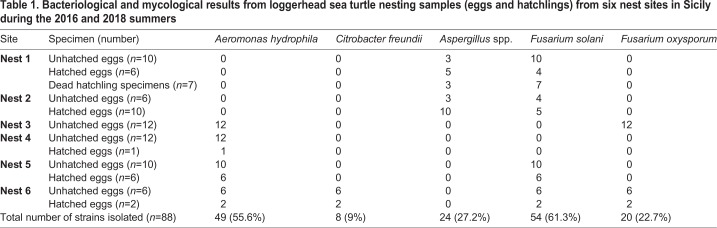
**Bacteriological and mycological results from loggerhead sea turtle nesting samples (eggs and hatchlings) from six nest sites in Sicily during the 2016 and 2018 summers**

Bacteriological analyses showed the absence of potential pathogens in all specimens from the first two nests (nests 1 and 2). However, 57 bacterial strains were isolated from the other four nests (nests 3, 4, 5 and 6), and these were biochemically identified as *Aeromonas hydrophila* (*n*=49) and *Citrobacter freundii* (*n*=8). Specifically, samples from nests 3, 4 and 5 were positive for the growth of *A. hydrophyla* (*n*=41), while from nest 6, both *A. hydrophyla* (*n*=8) and *C. freundii*. (*n*=8) were isolated (Table 1).

All samples, except those from nest 4 (from Linosa Island), were positive for one or more fungal colonies: in particular three types of morphologically different fungal colonies grew on Sabouraud Dextrose Agar (SDA). From nests 1 and 2, two different colonies ascribable to the *Aspergillus* genus were isolated (*n*=24). Although molecular identifications were not performed, the typical growth on SDA, associated with microscopic observations, suggested these colonies belonged to *A. fumigatus* and *A. flavus* species*.*

**Fig. 1.**
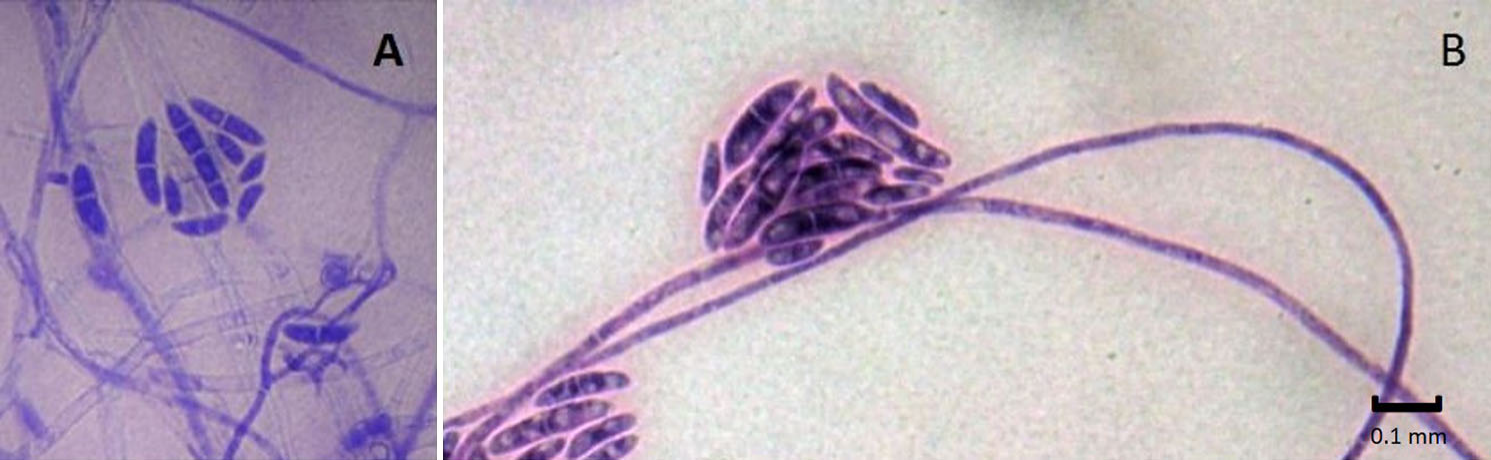
**Microscopic appearance of *Fusarium* spp. stained with Lactophenol Blue or Giemsa.** (A) Lactophenol Blue, (B) Giemsa. Magnification of 40×.

In addition, amongst the SDA plates (*n*=88), 71 showed fungal colonies potentially attributable to *Fusarium* spp. (Table 1); these colonies were white-cream and salmon pink, with a light brown reverse. The microscopic appearance supported a *Fusarium* genus identification and suggested the possible presence of two species: *F. solani* and *F. oxysporum* (Fig. 1) (Leslie et al., 2006; Nelson et al., 1983).

Morphological observations of these fungal colonies were confirmed by molecular analyses performed randomly on at least two positive plates from each nest. Sequences from the internal transcribed spacer (ITS) region were compared with data available from GenBank^®^ and confirmed the colonies were *F. solani* and *F. oxysporum*.

**Fig. 2.**
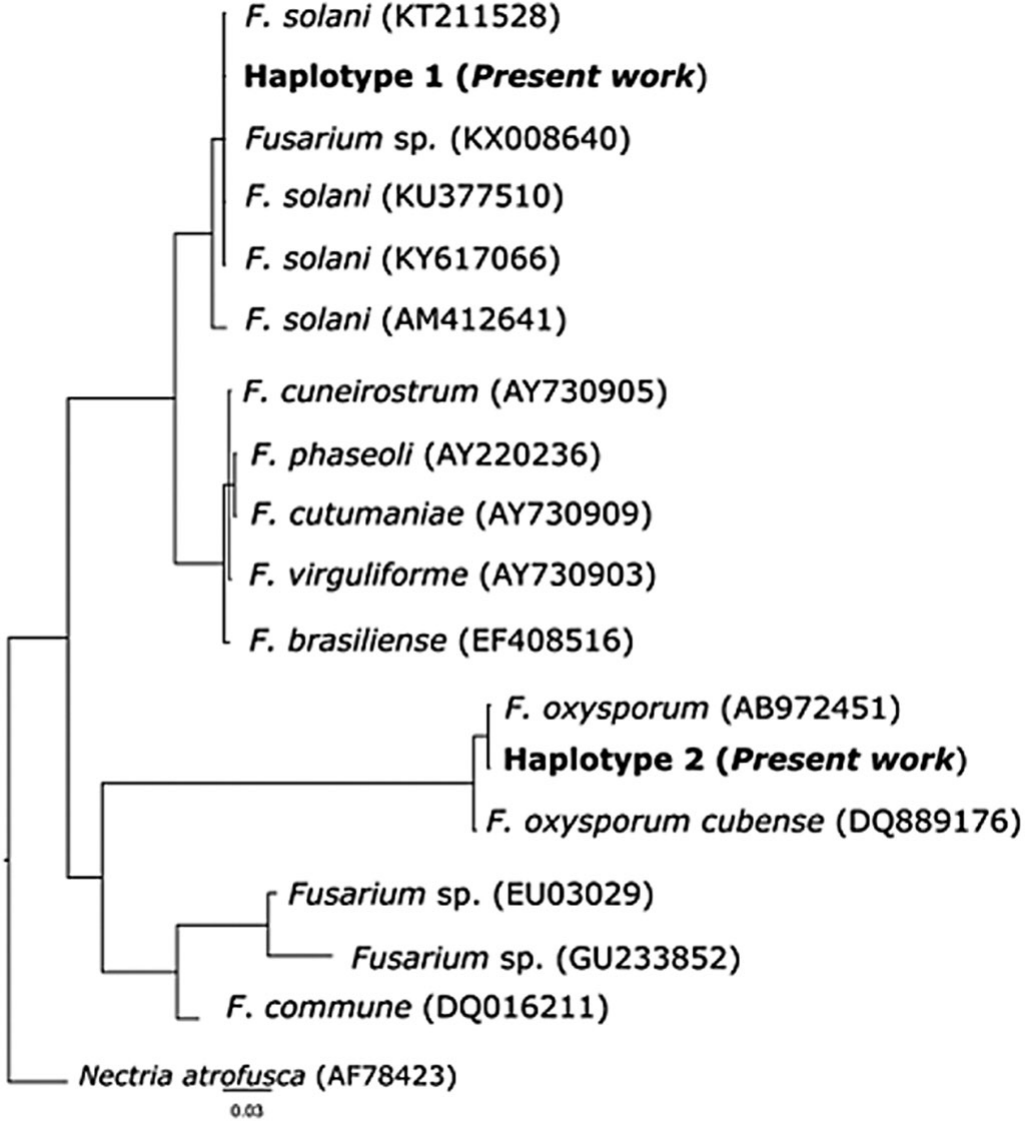
**Bayesian phylogram based on a 491-bp-long fragment of the ITS gene.** Node support is reported as ‘BI nodal posterior probabilities’/‘ML bootstrap support’. The accession numbers of the sequences derived from GenBank are shown in brackets. ‘Haplotype 1’ was observed in two nests (nests 3 and 6) and corresponds to *F.* *oxysporum*. ‘Haplotype 2’ was observed in four nests (nests 1, 2, 5 and 6) and corresponds to *F.* *solani*. See Table 1 for details on the occurrence of the two species in the studied sites and samples.

A single haplotype was observed for each of the two species (GenBank accession number MN960391–92), and these two haplotypes were identical to the haplotypes in GenBank^®^ for these species. Accordingly, all 71 sequenced colonies were ascribed to *F. solani* or *F. oxysporum* (Fig. 2 and Table 1).

PERMANOVA analyses on the multivariate dataset showed significant differences between nests and the presence/absence of bacteria (Si), whereas no significant differences were observed between the interaction eggs and sites [Eg (Si)] as shown in Table 2.

**Table 2.**
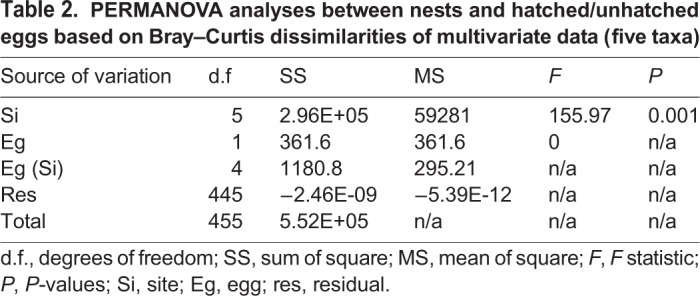
**PERMANOVA analyses between nests** **and hatched/unhatched eggs based on Bray–Curtis dissimilarities of multivariate data (five taxa)**

## DISCUSSION

For the first time, this study has investigated the microflora in eggs and dead hatchlings from six loggerhead sea turtle nests located on the Sicilian coast.

We detected bacterial strains belonging to the genera *Aeromonas* and *Citrobacter,* and fungi belonging to the genera *Aspergillus* and *Fusarium*. Specifically, the most prevalent microorganism strains were *Fusarium* spp. (80.6%) followed by *A. hidrophyla* (55.6%). Importantly, our results are in agreement with previous data on the microflora of sea turtle nests and on possible pathogens (bacteria and fungi) that influence hatching (Keene et al., 2014; Phillott and Parmenter, 2014; Sarmiento-Ramírez et al., 2010). Indeed, bacteria belonging to the *Aeromonas* genus are ubiquitous and are often isolated in marine and coastal environments (Dumontet et al., 2000; Fiorentini et al., 1998). *Aeromonas* spp. can infect eggs by penetrating shell pores, where they exploit interior substrates, allowing the bacteria to proliferate (Soslau et al., 2011). Although turtle shells are semi-permeable, they do not completely inhibit the passage of bacteria as eggs in contaminated substrates can acquire internal infections within hours of bacterial contact (Feeley and Treger, 1969). Wyneken et al. (1988) reported pathogenic microorganisms in the eggs of *C. caretta* that could explain the significant losses of hatched eggs occurring in sea turtle nests (Wyneken et al., 1988). Indeed, approximately 75% of bacteria isolated from turtle eggs can play pathogenic roles, and were also detected in mammals, amphibians, birds and fish (Craven et al., 2007). This could lead to the hypothesis that their roles are as opportunistic pathogens in turtle eggs when in conditions that favour proliferation.

Fungi from the *Fusarium* genus are considered saprophytes, but they can act as opportunistic pathogens in immunocompromised subjects or in developing embryos, especially under environmental stress conditions (Güçlü et al., 2010). Critically, the two *Fusarium* species identified in this study, *F. solani* and *F. oxysporum,* are recognised as causes of reduced hatching rates in sea turtle nests, and can occasionally cause 100% mortality in turtle embryos (Güçlü et al., 2010; Keene et al., 2014; Neves et al., 2015; Sarmiento-Ramírez et al., 2010). The ability of *F. solani* and *F. oxysporum* to penetrate egg shells and invade the embryonic tissue is due to the production of lipolytic and proteolytic enzymes that degrade inorganic and organic egg components (Phillott, 2004). Additionally, Bailey et al. (2018) demonstrated the presence of *Fusarium* DNA (*F. falciforme* and *F. keratoplasticum)* in embryonic fluid and biofilms from 73 fully incubated, unhatched loggerhead sea turtle eggs collected from different regions of North America. However, a recent molecular study from Turkey identified fungi from five genera (*Aspergillus*, *Emericella*, *Rhizopus*, *Actinomucor* and *Apophysomyces*) isolated from successfully hatched green turtle (*Chelonia mydas*) nests on eastern Mediterranean coasts (Candan, 2018). Moreover, these authors demonstrated that the hatching success of nests contaminated by fungi was significantly lower than those of uncontaminated nests.

Although this study does not sufficiently demonstrate the cause of *C. caretta* sea turtle nest failures on the Sicilian coastline, it does present key information on the microflora found in such nests with hatched/unhatched eggs and dead hatchlings. According to our observations on Linosa Island nests (using data loggers), the temperature and humidity recordings of these particular nests were very high during incubation periods. In particular, the average recorded temperatures were in the range 30–35°C, while the relative humidity was greater than 95% (unpublished data). These high values are potentially lethal for embryonic development and are optimal for the development of pathogenic microflora. Data loggers should be increasingly used to constantly record the main environmental variables (temperature and moisture) associated with the study of the characteristics/nature of the substrate. In this particular instance (Linosa Island), the granulometric composition of the beach where the eggs were laid may have had important roles in pathogen development. Interestingly, site diversification was highlighted by PERMANOVA analyses, suggesting a different contribution of this particular nest to the microflora. Environmental factors which influence hatching success, such as the different sand grain size at nesting sites, humidity, soil temperature and interference from anthropic activities should also be taken into consideration in future studies. Moreover, according to several studies, cloacal swabs should be taken immediately after egg laying to check if microflora has been transmitted to the young by the mother (Phillott, 2004; Phillott and Parmenter, 2014). Finally, our hypothesis that the hatching failures at our six nests were caused by pathogenic microorganisms requires more information and analysis.

Considering that in all the nests studied, the number of unhatched eggs was high, it is not surprising that *A. hydrophila*, *F. oxysporum* and *F. solani* were isolated. Besides being ubiquitous and widespread in marine and coastal environments, these microorganisms have been associated with hatching failures in the past (Phillott, 2004; Sarmiento-Ramírez et al., 2010). Furthermore, from our results table (Table 1), we observed that *A. hydrophila* occurred in association with *Fusarium* spp. in nests where the numbers of unhatched eggs exceeded hatched eggs. However, in the absence of cloacal swab analysis of the females, we cannot state for sure if these microorganisms were the cause of the hatching failures, or if these microorganisms proliferated by finding favorable substrates in the unhatched eggs.

This study only focused on six nests from four territorial sites, therefore wider investigations over extensive areas are required to better understand the causes of hatching failures, as well as the high hatchling mortalities caused by microorganism contamination or environmental conditions.

## MATERIALS AND METHODS

### Sample collection

As part of the monitoring activities of the Centro di Referenza Nazionale sul benessere, monitoraggio e diagnostica delle malattie delle tartarughe marine (CReTaM) of the Istituto Zooprofilattico Sperimentale della Sicilia (IZSSi), during the 2016 and 2018 summers, the centre received 463 samples from six different nests on the Sicilian coast (Table 3).

**Table 3.**
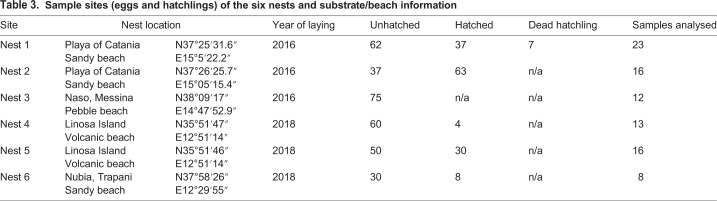
**Sample sites (eggs and hatchlings) of the** **six nests and substrate/beach information**

Samples were transferred to the IZSSi in sterile biological bags for laboratory analyses. Bacteriological and mycological analyses were performed for each nest, on hatched and unhatched eggs that were randomly selected. A total of 56 unhatched eggs, 25 hatched eggs and seven dead hatchlings were analysed (Table 3).

### Bacteriological and mycological analyses

All samples were washed in sterile water to remove sand residues. The shells from hatched eggs were homogenised in 9 ml alkalin pepton water (APW) broth and incubated at 25°C for 24–48 h. For unhatched eggs, after opening with sterile scissors and sterile forceps, swabs were taken from the shell and the contents. For dead hatchlings, swabs were taken from the belly surface, after which an incision was made with a sterile scalpel to allow swabbing of the abdomen. The swabs were then transferred to 9 ml APW and incubated at 25°C for 24–48 h. After this period, approximately 10 µl APW was aseptically spread onto selective agar plates. Samples were spread onto blood agar for the growth of different bacterial species and thiosulphate citrate bile salts sucrose agar for *Vibrio* spp isolation. These plates were incubated at 25°C for 24–48 h. Samples were spread onto McConkey agar plates for the isolation of *Enterobacteriaceae* and mannitol salt agar plates for the isolation of *Staphylococcus* spp. Plates were incubated at 37°C for 24 h. The presence of *Salmonella* spp. in egg contents and embryos (when present) were also tested. Firstly, samples for pre-enrichment were placed into 9 ml buffered peptone water broth, followed by enrichment in selenite cystine broth and Rappaport Vassiliadis broth, and then seeded on xylose lysine deoxycholate agar and brilliant green agar. After dissociation in generic culture medium, bacterial isolates were identified using the API test (Awong-Taylor et al., 2008).

Mycological examinations were conducted by seeding shells and swabs on SDA. Plates were incubated at room temperature for 7 days. After this period, the isolated fungi were stained with Giemsa and morphologically identified according to guidelines from Leslie et al. (2006) and Nelson et al. (1983).

### Molecular identification of fungus

Among the 71 SDA plates positive for *Fusarium* spp. growth, two positive plates from each nest were randomly selected for molecular identification. Fungal DNA was extracted using the Quick-DNA™ Fungal/Bacterial Miniprep Kit (Zymo Research, Irvine, CA, USA), according to the manufacturer’s instructions. PCR of the ITS region was performed using the primer pair; ITS-1 (5′-TCCGTAGGTGAACCTGCGG-3′) and ITS-4 (3′-TCCTCCGCTTATTGATATGC-5′) as described previously (White et al., 1990). PCR products were purified and sequenced by Macrogen Inc. (Seoul, South Korea) on an ABI3130xL (Applied Biosystems, Carlsbad, CA, USA) sequencer.

In addition to comparing our sequences with those available in the public repository, GenBank, 15 *Fusarium* spp. sequences and one *Nectria atrofusca* sequence were used as outgroups and included in these analyses (see Fig. 2 for accession numbers). Novel and GenBank sequences were aligned using ClustalX (Thompson et al., 1997) and manually trimmed to remove tails which were not present in all samples. The jModelTest ver 2.1.10 (Darriba et al., 2012) was used to test for the best fitting models of nucleotide substitution for the ITS dataset, under Akaike information criterion; the best-fit model proved to be a generalised time-reversible model with a gamma distribution rate variation among sites (GTR+G).

The genetic identification of our samples was performed using Bayesian inference (BI) and maximum likelihood (ML) methods as implemented in MrBayes v. 3.2.6 (Ronquist et al., 2012) and PhyMl v. 3 (Guindon and Gascuel, 2003), respectively. As a measure of branch support, bootstrap values were calculated with 1000 replicates in the ML trees. For the BI, two independent Markov Chain Monte Carlo analyses were run with 2 million generations (temp.: 0.2; default priors). Trees and parameter values were sampled every 100 generations resulting in 20,000 saved trees per analysis; an initial fraction of 5000 trees (25%) was conservatively discarded as ‘*burn-in*’. Nodes' statistical support of BI was evaluated by their posterior probabilities.

### Statistical analyses

A permutational multivariate analysis of variance (PERMANOVA, Anderson et al., 2008) was performed to test the null hypothesis referring to the presence/absence of different bacteria between nests and hatched/unhatched eggs. The analysis was based on Bray–Curtis dissimilarities (Bray and Curtis 1957) presence/absence dataset, and each term in the analysis was tested by 1999 random permutations of the appropriate units. The experimental design comprised two factors [Site (Si; six levels, fixed and orthogonal) and eggs (Eg; two levels, random and nested in Si)] and five variables – presence/absence of the following taxa: *A. hydrophila*, *C. freundii*, *Aspergillus* spp., *F. solani* and *F. oxysporum*.
